# Role of peer learning and self‐efficacy in parasympathetic activity during the simulation learning process in nursing students

**DOI:** 10.1002/nop2.1321

**Published:** 2022-08-14

**Authors:** Natsuki Nakayama, Harumi Ejiri, Naoko Arakawa, Tsuneko Makino

**Affiliations:** ^1^ Department of Integrated Health Sciences, Graduate School of Medicine Nagoya University Nagoya Japan; ^2^ Department of Nursing, College of Life and Health Sciences Chubu University Kasugai Japan

**Keywords:** nursing, parasympathetic nervous system, peer, self‐efficacy, simulation training, students

## Abstract

**Aims:**

This study aimed to investigate the stress level, including parasympathetic nervous activity, of students engaged in peer learning during simulations and the role of self‐efficacy.

**Design:**

Observational‐comparative study.

**Methods:**

The participants were 76 nursing students who were asked to evaluate a stable postoperative patient in Scene 1 and the same patient bleeding in Scene 2. In each scene, the students engaged in phases of repeated individual observations of the patient and discussions with peers. We compared each participant's parasympathetic activity during each observation in Scenes 1 and 2. Furthermore, the self‐efficacy score before the simulation was used to divide the participants into 3 groups, and the self‐efficacy and parasympathetic activity during the simulation were analysed.

**Results:**

The participants' parasympathetic activity decreased in both scenes with each phase of repeated observation. Two‐way repeated‐measures analysis of variance showed no statistically significant difference in parasympathetic activity during simulations among the three self‐efficacy groups.

## 1 INTRODUCTION

Simulations using high‐fidelity mannequins have recently been recommended as an interactive learning method. However, it has been suggested that more effective learning requires a focus on peer learning, stress control and self‐efficacy.

Nursing students who participate in educational programmes that use high‐performance simulators and virtual simulations can improve their critical thinking and clinical decision‐making skills (Hallin et al., 2016; Padilha et al., 2019).

Peer learning, on the contrary, is an educational process in which groups of students of the same age or with the same level of experience learn from each other. Compared with traditional supervision, peer learning is a useful way to significantly improve the self‐efficacy of nursing students (Pålsson et al., 2017).

In recent years, there has been a growing search for effective teaching methods that focus on general self‐efficacy. Self‐efficacy is defined as a belief in the opportunity of achieving an intended goal in a particular living situation (Bandura, 1997, 2001, 2009; Locke, 1987). In addition, self‐efficacy supports mental well‐being and active coping with stress (Bakker & Demerouti, 2008). Therefore, it is suggested that individual differences in self‐efficacy may have an effect on controlling stress in the simulation learning process for more effective simulation learning with peers.

### 1.1 Background

Cummings and Connelly (2016) and Li et al. (2019) showed that a simulation‐based deliberate practice programme is a viable teaching method aimed at improving the communication, empathy and self‐efficacy of nursing students. In addition, physiological stress has been shown to be associated with poorer performance during simulation, indicating that adaptive stress coping skills may enable people to manage stressful situations and maintain better performance (Anton et al., 2021). Despite the benefits of using high‐fidelity mannequins with a high degree of control over environmental conditions, the issue of stress, especially during simulations, continues to receive a great deal of attention. Boostel et al. (2018) measured stress before and after a simulation and found that stressors under conditions of a lack of competence and interpersonal relationships increased compared with that during normal laboratory learning. Therefore, it was suggested that stress in the simulation learning process needs to be controlled for more effective learning.

Carey et al. (2018) concluded that in peer learning, peer support alleviates clinical practice challenges, peers act as role models to enhance clinical knowledge, and support and feedback promote capacity and self‐confidence development and reduce stress and anxiety.

Carey et al. (2018) concluded that peer support reduces the challenges of clinical practice and that peers serve as role models to enhance clinical knowledge. In addition, peer support and feedback promote the development of competence and confidence and reduce stress and anxiety.

Curtis et al. (2016) showed that students who participated in student‐led simulations with peer learning reported high confidence in their nursing skills and high satisfaction with simulation learning. These previous studies suggest the effectiveness of combining simulation and peer learning. On the contrary, Nakayama et al. (2021) studied students' stress during simulation and observed a statistically significant increase in stress associated with a lack of competence, a lack of control over patient relationships, emotional involvement and contact with other students. Therefore, the presence of peers has been shown to influence students as a simulation‐specific stressor during peer learning.

Molero Jurado et al. (2019) stated that effective stress management is needed through more effective self‐efficacy enhancement programmes. Rayan (2019) showed that stress is negatively correlated with self‐efficacy and that psychosocial interventions to increase self‐efficacy might be beneficial for stress management. These previous studies suggest that we need to focus on stress control and self‐efficacy in peer simulation for more effective learning. Identifying strategies for understanding and managing the stress of nursing students is a challenge for nursing professors and can contribute to the safety of patients in the hospital.

The purpose of this study was to investigate the stress level, including parasympathetic nervous activity, of students engaged in peer learning during simulations and the role of self‐efficacy.

## 2 METHODS

### 2.1 Study design

This study was an observational and comparative study of students who participated in a peer‐learning simulation. The participants were assessed by changes in parasympathetic nerve activity.

### 2.2 Simulation scenarios

#### 2.2.1 Prebriefing

The simulation involved the use of a high‐fidelity manikin to simulate the patient (Laerdal Co., Ltd.). The participants were asked to assess a stable postoperative client in Scene 1 and when the same patient began to bleed in Scene 2. The researchers gave a simulation briefing before Scene 1 began, explaining the characteristics of the high‐fidelity manikin and giving the participants time to freely touch the manikin (10 min). Then, the researchers explained each phase of Scene 1 and Scene 2 to the participants. After this explanation and rest, the participants spent 5 min sitting in chairs in a space partitioned by curtains (Control).

#### 2.2.2 Simulation scenario

The simulated patient was a 53‐year‐old man who underwent partial gastrectomy for gastric cancer. In the simulation, Scene 1 immediately followed gastric cancer surgery on the patient, and Scene 2 involved intra‐abdominal bleeding on the first day after surgery. In Scene 1, the patient had completely recovered consciousness in the operating room, and his tracheal tube had been removed 1 h before the participants examined him. When the participants observed the patient, he was drowsy and wore an oxygen mask at a flow rate of 5 litres per minute. The patient had a central venous catheter, nasogastric tube, urethral catheter, bandages to protect the abdominal wound and an indwelling abdominal drain. The patient's vital signs were as follows: heart rate of 62–70 beats/min (bpm), blood pressure of 120–128/66–70 mmHg, respiratory rate of 16–20 breaths/min and oxygen saturation of 99%. Scene 2 was the first day after the patient's surgery; the patient's consciousness was clear, and he was in a private ward room after surgery. The patient was not receiving oxygen when the participants observed him. There was no difference in the patient's breath sounds between the left and right lungs, and his breathing was clear. According to the patient's blood test data, the haemoglobin level was 6.4, and the patient complained of abdominal pain. The patient's fingers were cold and showed cyanosis. The patient continued to have central venous catheters, nasogastric tubes, urethral catheters, bandages to protect abdominal wounds and indwelling abdominal drains. The participants found fresh blood in his abdominal drain bag, confirming an increase of 50 ml after 15 min. The participants could not hear the patient's intestinal peristalsis. The patient's vital signs were heart rate of 118–120 bpm, blood pressure of 78–80/40–44 mmHg, respiratory rate of 22–30 breaths/minutes and oxygen saturation of 90%. The researchers set the patient's condition as intra‐abdominal haemorrhage and set a 15‐min break between Scenes 1 and 2.

#### 2.2.3 Simulation structure

Scene 1 contained 5 phases. In Phase 1, the participants observed the patients individually (5 min). In Phase 2, the participants shared information with their peers (5 min). In Phase 3, each participant again observed the patient individually, and missing information was collected. In Phase 4, researchers gave time for each participant to reflect on what they had learned for independent learning (5 min). The participants then shared information with their peers in Phase 5 after summarizing their individual assessments (5 min). After that, the participants had a 15‐min break. The participants used the last 5 min of the 15 min as rest time (Rest). Scene 2 included four phases, that is Phases 6–9. In Phase 6, the participants observed the patient individually (5 min). In Phase 7, the participants shared information with their peers (5 min). In Phase 8, each participant again observed the patient individually, and missing information was collected (5 min). In Phase 9, the participants summarized the peer assessments and shared information with their peers (5 min).

#### 2.2.4 Debriefing

Finally, a debriefing was conducted with the participants and researchers (after 10 min). The debriefing was facilitated by the educator/researcher and was conducted by each group at the end of the simulation. The researcher actively engaged the participants, focused on learning and improvement and provided responses, commentary and analysis in accordance with Bajaj's recommendations for “promoting excellence and reflective learning in simulation” (Bajaj et al., 2018).

### 2.3 Participants

All third‐year students at two universities were invited to participate in the study during their respective final classroom sessions, and the study was explained to them. At the same time, the inclusion and exclusion criteria (heart disease, diabetes mellitus and taking medication on a daily basis) were explained, and the students were invited to participate. The participants in this study were third‐year undergraduates in a 4‐year course at one of two nursing universities. All participants had completed basic nursing courses, such as anatomy and physiology, and anatomy courses, such as skills and theory. None of the participants had experience with simulation using a high‐fidelity manikin. Therefore, the researchers prepared a simple scenario that the participants would easily manage. All participants were asked to avoid alcohol and caffeine the day before the study to avoid effects on their parasympathetic nervous systems. Among the students who participated in this simulation, those who agreed to the study were matched with peers and participated in this study. One group consisted of four to six people. The daily friendships and academic performances of the participants were not considered.

### 2.4 Data collection

**FIGURE 1:**
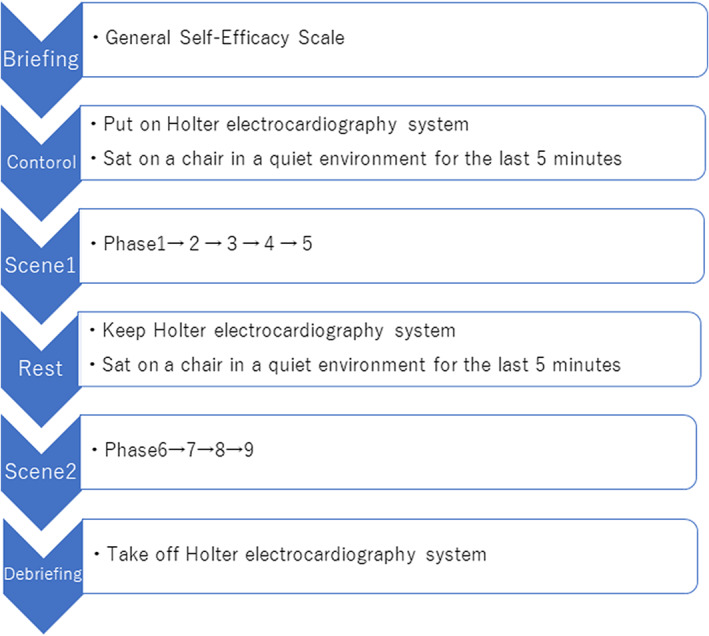
Data collection

All participants completed the Japanese version of the General Self‐Efficacy Scale (Sakano & Tohjoh, 1986). The researchers waited in another room while the participants completed the questionnaire. Then, the researchers connected each student to a Holter electrocardiography system (GLLERT Lab Tech Co., Ltd., Hungary). The researchers allowed the participants to rest for 5 min before beginning Phase 1 of Scene 1. The participants were asked to sit on a chair in a quiet environment and to relax by taking deep breaths (Figure 1).

### 2.5 General self‐efficacy scale

From the total score, the students' standard scores are used to rate their self‐efficacy on a five‐point scale to indicate the strength of their self‐efficacy: 1 = very low, 2 = low, 3 = normal, 4 = high and 5 = very high. The reliability and validity of this scale have been established in students. In the previous study, those with scores of 1 or 2 (≤40 points) were categorized into the low general self‐efficacy group, those with scores of 4 or 5 (≤65 points) were categorized into the high general self‐efficacy group, and those with a score of 3 were classified into the intermediate group.

### 2.6 Heart rate variability

The high‐frequency (HF) component of heart rate variability (HRV) reflects the activation of the vagus nerve, and the higher the value is, the greater the parasympathetic nerve activation (Task Force of the European Society of Cardiology the North American Society of Pacing Electrophysiology, 1996). Increased parasympathetic activity is associated with stress relief, and decreased parasympathetic activity is associated with increased stress (Hallin et al., 2016). In previous simulation studies, HRV has also been used to objectively evaluate stress status (Nakayama et al., 2018, 2021). Therefore, researchers can measure HRV to assess parasympathetic activity as a stress condition during student simulations.

Heart rate variability is the change in the R‐R wave interval in the measurement of successive normal electrocardiography (ECG) signals. Heart rate frequency analysis (MemCalc GMS) can be used to extract the HF component (HF: 0.15–0.4 Hz), which can be used as an indicator of parasympathetic activity (Punita et al., 2016; Sin et al., 2016). In the present study, we measured the participants' heart rates and HF components at the following simulation phases: Control, Phase 1, Phase 2, Phase 3, Phase 4, Phase 5, Rest, Phase 6, Phase 7, Phase 8 and Phase 9. We captured the changes in HR and HF components and clarified the changes in stress during the peer simulation. Furthermore, using the GSES, we divided the participants into a low general self‐efficacy group with scores of ≤40 points, an intermediate group and a high general self‐efficacy group with a score of ≤65 points, and changes in stress during the simulation in each of the three groups were determined.

### 2.7 Analytical methods

The data were compared using paired t‐tests in SPSS v.26 software (IBM Corp., Armonk, NY, USA). Scene 1 was compared with Control, and Scene 2 was compared with Rest to clarify the change in the HF component in the simulation with peer learning. In addition, in Scene 1 and Scene 2, we compared Phase 1 with Phase 3 and Phase 6 with Phase 8, in which the individual observations were repeated. The statistical significance level was set to *p* < .05. In addition, self‐efficacy scores were used to examine whether differences in self‐efficacy led to differences in parasympathetic activity during peer simulation. A two‐way repeated‐measures ANOVA was used to compare changes in the HF component in Scene 1 and Scene 2 among the low, intermediate, and high general self‐efficacy groups during the simulation. A *p*‐value of <.05 was considered statistically significant.

### 2.8 Ethical considerations

The researchers explained to all participants that participation was voluntary and that they could withdraw from the study at any time without affecting their grades. The researchers obtained informed consent from all the participants. This study was conducted with the approval of the research ethics committee of the university that conducted the study.

We adhered to the Strengthening the Reporting of Observational Studies in Epidemiology (STROBE) guidelines in the present study.

## 3 RESULTS

We invited 174 students from two universities to participate in our study. The present study enrolled 75 students with an average age of 20.8 years. There were 59 females (78.6%) and 16 males (21.3%) (Table 1).

**TABLE 1:** Characteristics

	All (*n* = 75)	GSES score		
		≤40 (*n* = 9)	41–64 (*n* = 53)	≥65 (*n* = 13)
Age, mean (SD)	20.8 (0.5)	21.0 (0.5)	20.9 (0.5)	20.7 (0.4)
Female, *n* (%)	59 (78.6)	6 (66.6)	43 (81.1)	10 (76.9)
Male, *n* (%)	16 (21.3)	3 (3.33)	10 (18.8)	3 (23.0)

Abbreviations: GSES: General Self‐efficacy Scale; SD: standard deviation.

**FIGURE 2:**
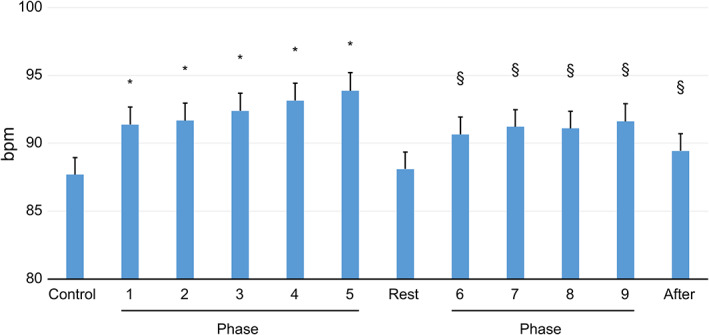
Changes in the mean (SE) heart rate in each phase. *p < .05, statistically significant difference vs. Control. §*p* < .05, statistically significant difference vs. Rest

During Scene 1 of the simulation with peer learning, heart rate increased significantly from Phases 1–5 compared with that during Control. In addition, in Scene 2, heart rate increased significantly from Phases 6–9 compared with that during Rest (Figure 2).

**FIGURE 3:**
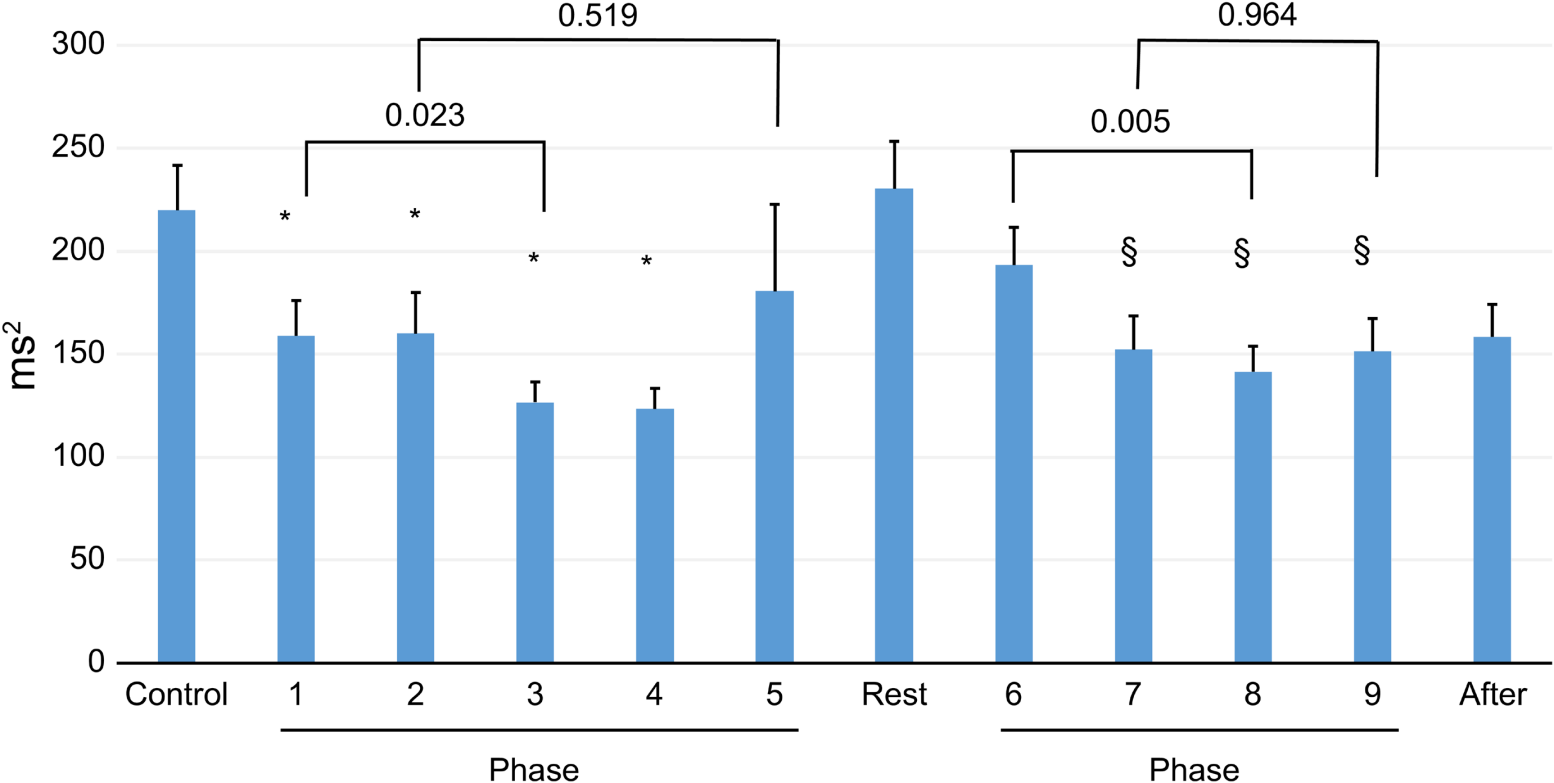
Changes in the mean (SE) high‐frequency heart rate variability in each phase. *p < .05, statistically significant difference vs. Control. §*p* < .05, statistically significant difference vs. Rest

In Scene 1, the HF component decreased significantly from Phases 1–4 compared with that during the Control. In addition, in Scene 2, the HF component decreased significantly from Phases 7–9 compared with that during Rest (Figure 3).

In a repeated comparison of the phases, in Scene 1, the HF component was significantly lower in Phase 3 than in Phase 1. Similarly, in Scene 2, the HF component was significantly lower in Phase 8 than in Phase 6. These phases were patient observation phases and decreased with each repetition of the observation in each scene. However, in the discussions with peers, there was no change in the HF component with repetition (Figure 3).

In the present study, we divided the participants into three groups by GSES score: ≤40 points (the low group), ≥65 points (the high group) and other (the intermediate group). The low group included 9 participants, 6 females and 3 males. There were 43 females and 10 males in the intermediate group. The high group included 10 females and 3 males (*p* = .610) (Table 1). The two‐way repeated‐measures ANOVA revealed no statistically significant difference in parasympathetic activity among the three groups in either Scene 1 or Scene 2 (*p* = .502, .356) (Table 2).

**TABLE 2:** Changes in the mean (SE) high‐frequency heart rate variability in each phase in each GSES group

	GSES score			*F*‐value	*p*‐value
	≤40 (*n* = 9)	41–64 (*n* = 53)	≥65 (*n* = 13)		
				0.695	.502
Control	183 (61)	225 (26)	231 (51)		
Phase					
1	108 (24)	160 (19)	192 (61)		
2	97 (22)	176 (27)	140 (26)		
3	92 (18)	136 (12)	115 (17)		
4	104 (21)	133 (13)	96 (16)		
5	92 (26)	184 (54)	120 (22)		
				1.049	.356
Rest	178 (48)	242 (30)	185 (38)		
Phase					
6	134 (25)	196 (22)	182 (40)		
7	110 (18)	146 (13)	133 (28)		
8	96 (18)	156 (16)	118 (21)		
9	119 (30)	162 (21)	135 (23)		
After	148 (45)	164 (20)	146 (22)		

Abbreviations: GSES, General Self‐efficacy Scale; SD, standard deviation.

## 4 DISCUSSION

The present study is the first report to clarify changes in students' stress during simulation with peer learning from the viewpoint of parasympathetic activity, as measured by HRV, and to discuss this topic from the perspective of general self‐efficacy.

In the present study, the HF component decreased significantly in the 3rd phase compared with that in the 1st phase and decreased in the 8th phase compared with that in the 6th phase during repeated individual patient observations. The participants engaged in discussion with peers after the 1st phase and 6th phase of patient observation. A low HF component indicates a decrease in parasympathetic activity and an increase in stress (Kim et al., 2018). Nakayama et al. (2021) showed that in face‐to‐face interactions involving vital sign measurements, the presence of other students increased stress. In the present study, the finding of increased stress in the post‐discussion observation with peers suggests that for the participants, the discussion with their peers might have led to the stress of the next patient observation phase. Carr et al. (2016) showed that the participants in the peer‐learning condition experienced deeper learning through teaching and learning from their peers and became more positive about giving and receiving feedback. Doherty‐Restrepo et al. (2018) showed that students said that reports provided by peers were as effective as reports from teachers. These previous studies showed that stress did not always impede learning for students. This study suggests that the lack of an effect of stress in impeding learning might be based on the positive effects of parasympathetic activity, as increased stress and increased concentration are associated with a decrease in the HF component. Therefore, compared with the first observation, the second observation might have partially contributed to the practical application of phased learning in discussion with peers not only by increasing stress but also by having a positive effect, such as keeping tension on the parasympathetic nervous system.

In contrast, the HF component did not change significantly between the 2nd and 4th phases or between the 7th and 9th phases. These results indicate that the students' stress did not change in repeated discussions with their peers. In a previous study, Nakayama et al. (2018) showed that the stage when the participants reported the patient's condition to the educator was characterized by high objective and subjective stress. In contrast, an observational study of peer learning among arbitrarily paired nursing students revealed that they could “practice nursing skills and abilities when working together,” “establish knowledge by understanding each other” and “share thoughts, knowledge and feelings” (Pålsson et al., 2021). Furthermore, Kaur et al. (2020) investigated the perceptions and experiences of stress among nursing students and concluded that nursing students determined how to understand and reduce the effects of stress through the value of self‐learning, self‐knowing and social support. Additionally, Christiansen and Bell (2010) suggested that active support from peers reduced the social isolation experienced by novice students. These studies have shown that learning with peers can help students control stress, establish knowledge and practice their skills. In addition to these previous studies, in the present study, we showed that repeated discussions with peers had fewer parasympathetic effects, suggesting that this parasympathetic activity supported knowledge establishment and skill learning.

The results of this study showed no clear difference in the change in stress during the simulation due to a difference in self‐efficacy. In previous studies, Jahanpour et al. (2010) showed that low self‐efficacy and stress experiences had a negative impact on clinical decision‐making. In addition, Zhao et al. (2015) suggested that high self‐efficacy had a positive effect on reducing stress and strategically working on problem solving. These previous studies suggest that differences in individual student self‐efficacy may affect changes in stress during a simulation. However, in this study, by performing simulations with peers, the difference in self‐efficacy did not affect the change in stress during the simulation. Carey et al. (2019) reported that the effects of the presence of peers were “connection with peers” and “cooperative support for advice and guidance.” In addition, Ravanipour et al. (2015) showed that nursing students reported general satisfaction with peer learning because it was less stressful and much more detailed than traditional learning methods. These previous studies suggested that the presence of peers may help keep students stressed and in a better learning state during simulations. In the present study, simulations using peer learning suggested that parasympathetic activity was controlled for step‐by‐step learning during the simulation, regardless of differences in self‐efficacy.

### 4.1 Limitations

Despite these new findings, this study has limitations. Since the peer groups were not organized in advance according to different levels of self‐efficacy, the influence of the self‐efficacy of the group members could not be considered.

## 5 CONCLUSION

The present study shows that repeated discussions with peers have less parasympathetic influence, suggesting that this parasympathetic activity supports knowledge establishment and skill learning. Therefore, compared with the first observation, the second observation might have partially contributed to the practical application of phased learning in discussion with peers not only by increasing stress but also by having a positive effect, such as maintaining tension on the parasympathetic nervous system. In addition, in the present study, simulations using peer learning suggested that parasympathetic activity was controlled for step‐by‐step learning during the simulation, regardless of differences in self‐efficacy.

## Data Availability

The data that support the findings of this study are available from the corresponding author, N. N, upon reasonable request. Because we are planning to use these data for other scientific themes, we cannot share these data here at the present time.
